# Recent advances in postoperative delirium in elderly patients: pathophysiological mechanisms, risk prediction, and therapeutic strategies

**DOI:** 10.3389/fnins.2026.1759910

**Published:** 2026-02-06

**Authors:** Xuan-yin Ding, Ming-hui Zhang, Jun Liu, Dan Wu

**Affiliations:** 1 Department of Anesthesiology, Zigong Fourth People's Hospital, Zigong, China; 2 Department of Neurosurgery, Zigong Fourth People's Hospital, Zigong, China; 3 Department of Radiotherapy, The Affiliated Hospital of Southwest Medical University, Luzhou, China

**Keywords:** elderly patients, pathophysiological mechanisms, perioperative management, postoperative delirium, risk factors

## Abstract

Postoperative delirium (POD) is a common acute neuropsychiatric syndrome occurring during the perioperative period, characterized by disturbances in consciousness, attention, and cognition. It frequently develops in elderly patients as a consequence of surgery-induced neurofunctional impairment and physiological stress. Clinically, POD manifests with an abrupt onset, fluctuating course, and symptoms such as confusion and disorganized thinking. Multiple predisposing and precipitating factors contribute to its occurrence, including preoperative mental status, general physical condition, diabetes mellitus, electrolyte imbalances, type and duration of surgery, intraoperative blood loss, anesthesia management, and medication use. Understanding the underlying mechanisms of POD in older adults and implementing targeted preventive and therapeutic interventions are crucial for reducing its incidence, enhancing perioperative recovery, and improving the overall safety of surgical care in the elderly population.

## 1 Introduction

**Figure 1:**
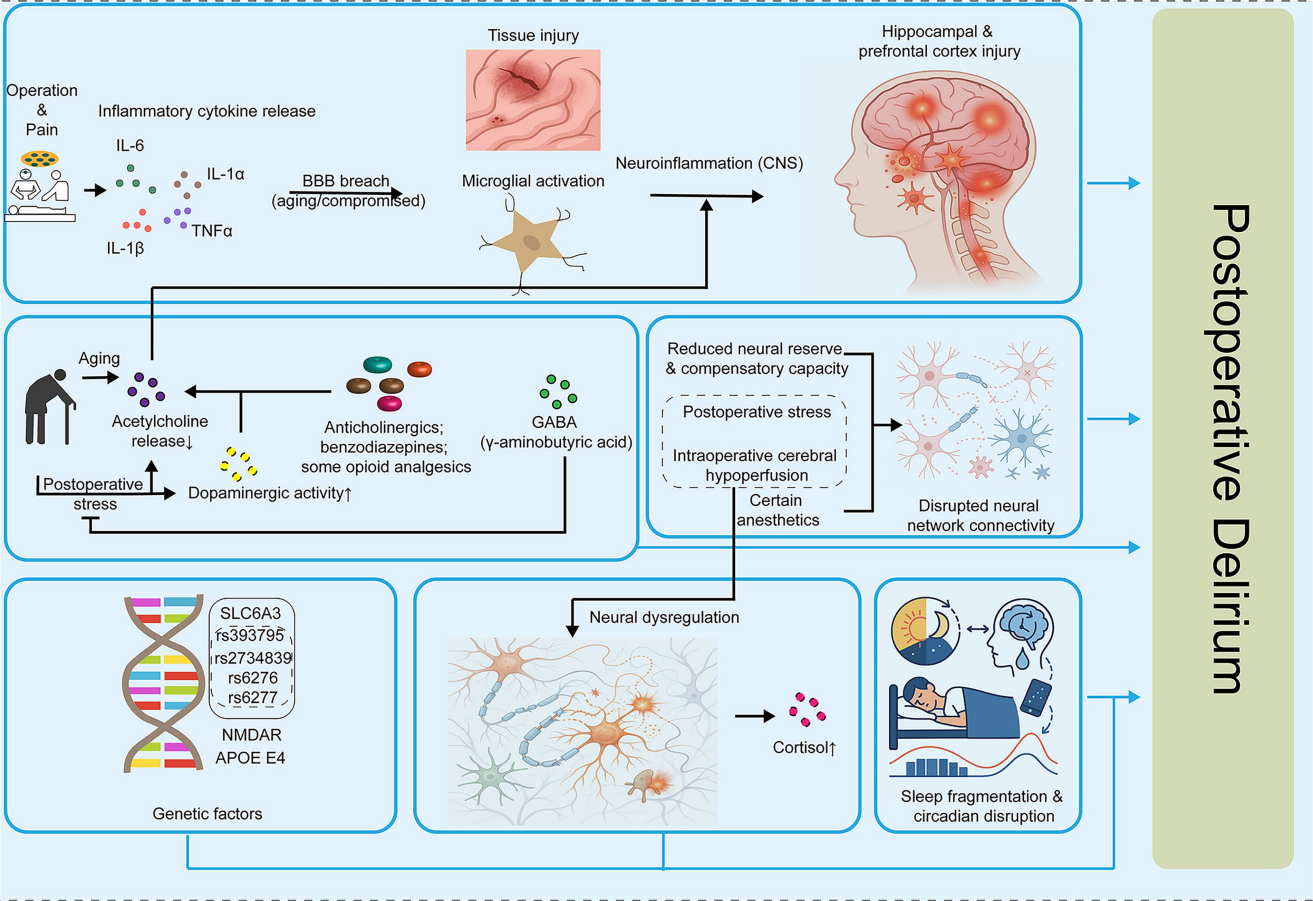
Mechanisms contributing to POD. Postoperative stress and pain trigger systemic inflammatory responses, releasing cytokines such as IL-1α, IL-1β, IL-6, and TNFα, which may compromise the blood–brain barrier (BBB) and activate microglia, leading to neuroinflammation and injury in hippocampal and prefrontal cortex regions. Aging and perioperative factors, including anticholinergic drugs, benzodiazepines, opioids, and altered dopaminergic or GABAergic activity, reduce neural reserve and compensatory capacity, disrupting neural network connectivity. Genetic predispositions (e.g., variants in SLC6A3, NMDAR, APOE ε4) further influence susceptibility. These combined insults result in neural dysregulation, elevated cortisol levels, and sleep fragmentation or circadian disruption, collectively contributing to the onset of POD. Arrows indicate causal or modulatory relationships between factors.

Postoperative delirium (POD) is an acute-onset neuropsychiatric syndrome and has become one of the most common complications among elderly surgical patients. Clinically, POD is characterized by acute and nonspecific disturbances of consciousness, altered mental status, impaired attention and cognition, and disruptions in the sleep–wake cycle. It typically develops during anesthesia recovery or within 1–3 days after surgery (Wilson et al., 2020; Boord et al., 2021). Based on clinical manifestations, POD can be categorized into three subtypes: hyperactive, hypoactive, and mixed. Approximately 50% of cases are hypoactive, presenting with lethargy, reduced speech, cognitive impairment, and apathy; hyperactive and mixed forms account for about 25% each, with the hyperactive subtype often involving agitation, hallucinations, and even aggressive behavior (Zhang et al., 2022). With the rapid progression of global population aging, the number of elderly patients requiring surgical intervention continues to rise, posing increasing challenges to the recognition, prevention, and management of POD. Hypoactive delirium is particularly prone to underdiagnosis and delayed treatment, which contributes to worse outcomes and increased mortality. Epidemiological studies report that the incidence of POD after major surgery in patients over 60 years of age ranges from 20 to 40% (Jin et al., 2020). Among general surgical patients, the incidence is approximately 13–41%, while in elderly individuals undergoing cardiac surgery or treated in intensive care units (ICUs), the incidence may reach 55% or even higher (Chen et al., 2021). POD can lead to multiple postoperative complications, significantly prolong hospitalization, and increase healthcare costs. Moreover, it is associated with long-term cognitive decline, impaired daily functioning, and elevated mortality risk (Ostertag et al., 2024). The development of POD is multifactorial, involving both patient-related and perioperative variables, such as advanced age, preoperative anxiety or depression, pre-existing cognitive impairment, stroke history, diabetes mellitus, metabolic or electrolyte disturbances, cardiopulmonary or cerebrovascular diseases, as well as intraoperative factors including surgical type and duration, blood loss, anesthesia techniques, medication choices, and postoperative analgesic regimens (Shang et al., 2024). Current evidence suggests that the pathophysiological mechanisms underlying POD may involve neuroinflammation, neurotransmitter imbalance, neural network dysfunction, and circadian rhythm disturbances (Farag et al., 2020). Despite growing research efforts, the exact mechanisms, predictive markers, and effective interventions for POD remain incompletely understood. This review aims to summarize recent advances in the study of POD in elderly patients, focusing on its pathophysiological mechanisms, risk prediction, and therapeutic strategies, thereby providing insights for clinical management and future research (see Figure 1).

## 2 Pathophysiological mechanisms of postoperative delirium

### 2.1 Neuroinflammation and blood–brain barrier disruption

Recent studies have highlighted the central role of neuroinflammation in the development of POD. During the aging process, elderly individuals exhibit reduced neural connectivity, enhanced neuroinflammatory responses, altered microglial function, and cerebrovascular dysfunction (Xiao et al., 2023). Surgical trauma, anesthesia, and postoperative pain can activate the peripheral immune system through multiple pathways, resulting in the excessive release of proinflammatory cytokines such as interleukin (IL)-1β, IL-6, IL-8, IL-10, C-reactive protein (CRP), and tumor necrosis factor-*α* (TNF-α) (He et al., 2021; Peng et al., 2023). These inflammatory mediators can penetrate the BBB, which becomes increasingly permeable with aging or systemic disease, thereby triggering microglial activation and initiating a cascade of neuroinflammation (Şaşkin et al., 2022). Clinical and experimental evidence supports this mechanism. Patients with delirium often show elevated leukocyte counts, increased neutrophil percentages, and a significantly higher neutrophil-to-lymphocyte ratio compared with non-delirious patients (Thisayakorn et al., 2021). Neuroimaging studies further demonstrate that such inflammatory responses preferentially damage brain regions responsible for higher cognitive functions and attention regulation, including the hippocampus and prefrontal cortex, contributing to postoperative cognitive disturbances. Cytokines can disrupt neuronal communication by altering neurotransmission, inducing neuronal apoptosis, and activating glial cells such as microglia and astrocytes. This leads to excessive production of reactive oxygen species (ROS), complement activation, glutamate release, and nitric oxide generation, all of which culminate in neuronal injury. Age-related BBB dysfunction and microglial sensitization further exacerbate these effects, rendering older adults more vulnerable to neuroinflammatory insults and neuronal apoptosis—a key explanation for the markedly higher incidence of POD in this population under similar surgical conditions (Taylor et al., 2022). In addition, central nervous system (CNS) inflammation has been shown to suppress excitatory synaptic transmission and activate the hypothalamic–pituitary–adrenal (HPA) axis, thereby increasing monoamine turnover. Elevated levels of norepinephrine, serotonin, and dopamine, together with reduced cerebral blood flow and increased cerebral oxygen consumption, can further precipitate the onset of POD (Huang et al., 2021). Numerous studies have confirmed that patients with POD exhibit significantly higher plasma cortisol levels than those without delirium (Cerejeira et al., 2013). Excess cortisol may induce neuronal dysfunction by impairing synaptic activity, promoting the release of inflammatory mediators, enhancing oxidative stress, destabilizing the cytoskeleton, and inhibiting glucose transport into neurons, collectively contributing to postoperative neurocognitive decline.

Notably, surgical stress can induce mitochondrial dysfunction and dysregulation of mitophagy, which in turn activates the NLRP3 inflammasome, triggering microglia-mediated neuroinflammation and ultimately contributing to the onset of postoperative delirium (Jia et al., 2024). The excessive generation of ROS and other proinflammatory mediators further amplifies this neuroinflammatory cascade (Zhou et al., 2024). Magnetic resonance imaging (MRI) studies have revealed that white matter lesions caused by BBB disruption during systemic inflammation are closely associated with the development of POD (Pendlebury et al., 2024). Emerging evidence also suggests that gut microbiota dysbiosis is strongly linked to POD through the gut–brain axis (Zhang et al., 2023). A reduction in gut microbial metabolites such as short-chain fatty acids may impair microglial maturation and disturb neurotransmitter balance, thereby indirectly aggravating neuroinflammation (Kalyanaraman et al., 2024). Currently, three major pathways are thought to underlie the initiation of neuroinflammation: (1) alterations in neurotransmission that promote inflammatory signaling within the CNS; (2) the translocation of peripheral cytokines across a compromised BBB, directly affecting neuronal communication; and (3) the activation of peripheral sensory nerves—particularly the vagus nerve—which stimulates cytokine release from central glial and neuronal cells (Yang et al., 2017). Clinical studies have demonstrated that elevated serum concentrations of IL-6 and soluble interleukin-2 receptor (sIL-2R) are positively correlated with the risk of developing POD (Noah et al., 2021), whereas levels of anti-inflammatory mediators such as IL-1 receptor antagonist and cerebrospinal fluid FMS-like tyrosine kinase-3 are inversely associated with its occurrence (Westhoff et al., 2013). These findings indicate that dysregulation of cytokine-based biomarkers plays a crucial role in the neurodegenerative processes underlying delirium. Furthermore, patients with POD are more likely to develop secondary complications such as sepsis, prolonged hospitalization, and increased mortality (Lee et al., 2023).

### 2.2 Neurotransmitter imbalance hypothesis

A large body of evidence suggests that abnormalities in central neurotransmitters such as acetylcholine, dopamine, serotonin (5-HT), *γ*-aminobutyric acid (GABA), and norepinephrine may be closely associated with the development of POD (Adam et al., 2020). With advancing age, both pathological and physiological brain aging lead to morphological alterations, neuronal loss, neuronal shrinkage, and reduced cerebral metabolism. Studies have shown that acetylcholine synthesis decreases in older adults, whereas its hydrolysis remains relatively unchanged. Under surgical stress, the combination of reduced acetylcholine synthesis and increased dopaminergic activity constitutes a critical mechanism predisposing elderly patients to delirium. Moreover, disruption of cholinergic homeostasis can exacerbate neuroinflammation, further contributing to the onset of POD (Dunne et al., 2021). Perioperative medications such as anticholinergics (e.g., atropine, glycopyrrolate), benzodiazepines, and certain opioids may aggravate this imbalance. Anticholinergic drugs, often used to reduce glandular secretions, can easily disrupt neurotransmitter equilibrium in elderly patients due to their already diminished central acetylcholine levels, thereby inducing postoperative delirium even at low doses (Gray and Hanlon, 2016). Clinical evidence indicates a dose-dependent association between reduced perioperative anticholinergic use and lower delirium severity. Notably, morphine metabolites can bind to central cholinergic receptors, directly impairing cholinergic transmission. Molecular imaging studies have revealed abnormally elevated acetylcholinesterase activity and upregulated dopamine D2 receptor expression in the brains of patients with POD. This bidirectional neurotransmitter imbalance reduces neural network efficiency, manifesting as cognitive slowing and fluctuating consciousness. Dopamine, an excitatory neurotransmitter in the central nervous system, suppresses acetylcholine release, which aggravates the hyperactive subtype of postoperative delirium (Hshieh et al., 2008). Furthermore, elevated plasma anticholinergic activity not only correlates with the occurrence of delirium but also with its severity. High acetylcholine levels can mitigate excessive inflammation, whereas cholinergic deficiency may promote neuroinflammation and exacerbate delirium symptoms (Sl and Hl, 2022). Beyond acetylcholine, imbalances in other neurotransmitters—such as increased norepinephrine and dopamine release, coupled with decreased GABA, serotonin, and histamine—have also been implicated in POD, although their precise mechanisms remain unclear. GABA, a key inhibitory neurotransmitter, attenuates stress responses and neural excitability, playing an essential role in balancing excitation and inhibition during delirium pathogenesis (Mulkey et al., 2018). Serotonin, a major modulator of cognition, arousal, and mood, has been reported to decrease in hyperactive delirium but increase in hypoactive forms (Maldonado, 2013). Selective serotonin antagonists can activate the PI3K/Akt/mTOR pathway, reduce IL-1 concentrations, and partially reverse delirium symptoms (Qiu et al., 2016). Dopamine excess can induce excitatory delirium by directly stimulating neuronal activity, enhancing glutamate-mediated neurotoxicity, and promoting oxidative stress–induced neuronal apoptosis, leading to network dysfunction and POD development (Liu et al., 2024). Taken together, these findings indicate that multi-neurotransmitter imbalance constitutes a fundamental mechanism underlying postoperative delirium.

### 2.3 Disruption of neural network connectivity

Advanced functional magnetic resonance imaging (fMRI) studies have revealed widespread dysfunction in neural networks among patients with POD (Noah et al., 2021). Resting-state analyses indicate that elderly patients exhibit weakened intra-network connectivity within the default mode network (DMN) after surgery, particularly between the posterior cingulate cortex and prefrontal regions, a disconnection closely associated with attentional deficits (Zhou et al., 2025). In contrast, hyperactivity of the salience network (SN) disrupts the ability to filter information, resulting in impaired processing of environmental stimuli. Due to reduced neuronal reserve and diminished compensatory capacity, the aging brain is particularly vulnerable to network connectivity disruption under surgical stress (Zhou et al., 2025). Diffusion tensor imaging (DTI) has further demonstrated that POD patients exhibit compromised connectivity across multiple regions, including the cerebral hemispheres, fronto-thalamo-cerebellar circuits, limbic structures, and memory-related networks. Intraoperative events such as hypotension or hypoxemia can exacerbate cerebral hypoperfusion, and when regional cerebral oxygen saturation (rSO₂) falls below 60%, the risk of postoperative neurocognitive complications in elderly patients increases significantly (Ding et al., 2023). Certain anesthetic agents may also induce connectivity alterations or disruptions across various cortical areas, contributing to delirium onset. Neural connectivity impairments in POD are also reflected in electrophysiological changes, such as increased slow-wave activity in the frontal and occipital lobes (Tanabe et al., 2020). Additionally, acute inflammatory responses have been reported to affect the function of central neurons and synapses, leading to functional segregation within neural networks (Zhu et al., 2023). Collectively, these findings suggest that disruption of large-scale neural network connectivity constitutes a key pathophysiological mechanism underlying POD in elderly surgical patients.

### 2.4 Genetic hypothesis

Several studies have indicated that genetic variations may influence susceptibility to POD. For example, the AA genotype at rs393795 of the dopamine transporter gene (SLC6A3) appears to confer a protective effect against delirium, whereas rs2734839, rs6276, and rs6277 are associated with increased POD risk (Sultan, 2010). Seven single nucleotide polymorphisms (SNPs) within SLC6A3 can modulate dopamine transporter function, leading to reduced dopamine availability in the brain and thereby contributing to delirium development (van Munster et al., 2010). Additionally, the AG haplotype of the N-methyl-D-aspartate receptor 3A (NMDA3A) gene has been reported to correlate with POD occurrence (Lin et al., 2013). The apolipoprotein E (APOE) E4 allele is associated with both the incidence and duration of postoperative delirium in elderly patients (Kunicki et al., 2023), and APOE E4 in combination with the BCHE-K gene may further relate to cognitive decline. Moreover, polymorphisms in the MTNR1B gene, which encodes the melatonin receptor MT2, have been linked to increased susceptibility to POD (Terrelonge et al., 2022). These findings collectively suggest that genetic predisposition, particularly involving neurotransmitter regulation, lipid metabolism, and circadian rhythm pathways, may play a significant role in the pathophysiology of POD.

### 2.5 Stress response

Perioperative stressors, including hypoxemia, cerebral hypoperfusion, cortical injury, and hypovolemia, can disrupt central nervous system homeostasis and provoke pathological elevations in cortisol levels. Such neuroendocrine dysregulation is considered a contributing factor to the development of POD in elderly patients.

### 2.6 Sleep–wake cycle

Disruption of sleep and circadian rhythm dysregulation have been identified as significant risk factors for POD (Leung et al., 2023). Melatonin, a hormone produced by the pineal gland, plays a critical role in regulating the sleep–wake cycle and maintaining circadian rhythm homeostasis (Jiang et al., 2023). Preoperative supplementation with exogenous melatonin has been shown to prevent POD in elderly patients, likely by mimicking natural circadian rhythms. Ramelteon, a melatonin receptor agonist commonly used for insomnia, has also demonstrated efficacy in reducing the incidence of POD (Han et al., 2020). Melatonin exhibits neuroprotective effects through multiple mechanisms, including anti-amyloid activity and modulation of the AMPK/CREB signaling pathway, thereby counteracting oxidative stress induced by brain injury, mitigating neuroinflammation, and attenuating neurofunctional decline, which collectively contribute to POD prevention (Jiang et al., 2023). Furthermore, numerous studies have confirmed that melatonin receptor agonists can effectively prevent POD in elderly surgical patients (Fazel et al., 2022; Mohamed et al., 2022; Tanifuji et al., 2022).

## 3 Identification of risk factors

### 3.1 Preoperative factors

Advanced age and frailty have been consistently recognized as key risk factors for POD (McIsaac et al., 2020). Studies indicate that the incidence of POD in patients aged 61–80 years is approximately 40% (Fenta et al., 2025), whereas patients over 80 years demonstrate a markedly higher incidence, showing a clear age-dependent increase. This heightened susceptibility is associated with age-related declines in cerebral autoregulation, reduced cerebral microvascular density, impaired vascular regeneration, decreased vascular density, and cerebrovascular lesions such as infarcts, all of which reduce cerebral perfusion and lower the threshold for neuroinflammation. Neuronal loss and diminished cerebral oxygen supply lead to decreased acetylcholine synthesis, while structural brain changes and cognitive decline further exacerbate vulnerability. Aging also promotes increased cytokine secretion, and when combined with anesthetic exposure and surgical stress, these factors collectively elevate the risk of POD in elderly patients. Frailty has been shown to reduce muscle mass and impair cognitive function, further increasing the likelihood of postoperative delirium (Shen et al., 2023). Another critical preoperative predictor is baseline cognitive status. Assessment using the Mini-Mental State Examination (MMSE) demonstrates that lower preoperative MMSE scores are associated with a higher incidence of POD, and patients with preexisting cognitive impairment are particularly susceptible (Bao et al., 2023). The MMSE is a standardized clinical tool widely used to evaluate mental status, with a dementia screening cutoff of ≤26 for individuals with at least a primary school education (Huo et al., 2021). The recent introduction of the concept of “perioperative frailty” has significantly enhanced risk prediction accuracy. A research team from Southern Medical University developed a 32-item multidimensional frailty index (FI-PGA) integrating nutritional status, comorbidities, and activities of daily living, which can independently predict postoperative cardiovascular events and POD risk. When combined with the NT-proBNP biomarker, the predictive net benefit is further improved (Xie et al., 2025). Most patients undergo preoperative fasting and fluid restriction for durations that frequently exceed the 2-h window recommended by clinical guidelines. Prolonged preoperative fasting and fluid restriction can induce dehydration in elderly patients, with subsequent metabolic disturbances leading to hypoglycemia and electrolyte imbalances. Hypoglycemia is associated with an increased risk of neurological manifestations including confusion, irritability, and delirium. Furthermore, extended fasting and fluid restriction may precipitate hemodynamic instability and intestinal dysfunction, which in turn disrupt the gut microbiota. Such dysbiosis can impair cerebral function via the gut-brain axis and elevate susceptibility to other postoperative complications. Accumulating evidence has indicated that preoperative fasting and fluid restriction exceeding 6 h is linked to a significantly higher risk of postoperative delirium. In an international observational cohort study of 1,002 patients, the incidence of delirium was markedly higher in patients with preoperative fasting and fluid restriction durations over 6 h than in those with durations of 2–6 h (12.9% vs. 5%, *p* < 0.001) (Radtke et al., 2010).

Preoperative infections, malnutrition, and acute vascular diseases can lead to cerebral ischemia and reduced cerebral oxygen supply, thereby causing brain dysfunction and increasing the risk of POD. A study involving 35,743 patients revealed that the incidence of postoperative delirium was 4.2% (1,519 cases) in patients with Parkinson’s disease and 2.3% (828 cases) in those without Parkinson’s disease (*p* < 0.001), indicating that patients with preoperatively comorbid Parkinson’s disease carry a higher risk of developing postoperative delirium (Dham et al., 2023), and those with preoperative dementia have an elevated risk of developing POD. Moreover, studies have indicated that patients who experience POD are at higher risk of subsequent dementia compared to those without POD, suggesting a bidirectional relationship in which dementia serves as a risk factor for POD, while POD itself also increases the risk of cognitive decline. Extensive evidence further demonstrates that preexisting conditions such as hypertension, diabetes mellitus, pulmonary infections, cor pulmonale, chronic obstructive pulmonary disease (COPD), stroke, atrial fibrillation, malnutrition, multi-organ dysfunction, anemia, elevated serum creatinine, carotid artery stenosis, sensory deficits (visual or auditory impairment), and excessive alcohol consumption are all significant risk factors for POD (Greaves et al., 2020). Toxins such as urea and ethanol can damage neurons, impair intercellular signal transmission, and reduce the brain’s capacity to process information from non-cortical regions, thereby contributing to POD. Postoperative use of statins has also been reported to increase the risk of POD. Preoperative anxiety, depression, and low educational attainment are additional risk factors for POD (Falk et al., 2021; Ren et al., 2021). Anxiety prior to surgery can impair postoperative pain control and promote the development of delirium and other complications. Mechanistically, preoperative anxiety may reduce presynaptic GABA release and inhibit postsynaptic GABA receptor function, exacerbating postoperative pain (Friedrich et al., 2022). Sleep disturbances before surgery can reduce hippocampal neuron numbers and disrupt neurotransmitter signaling in the central nervous system, leading to impaired memory and spatial cognition, which further increases POD susceptibility (Oldham et al., 2021). Preoperative vitamin D deficiency is another important risk factor. Vitamin D is widely distributed in brain regions critical for cognitive function, including the cerebral cortex, hippocampus, and hypothalamus (Hung et al., 2022). It exerts neuroprotective effects by modulating acetylcholine and dopamine levels, reducing C-reactive protein concentrations, and attenuating neuroinflammatory responses (Wang et al., 2023; Zhou and Hyppönen, 2023). Deficiency of vitamin D diminishes these protective mechanisms, rendering neurons more vulnerable to surgical stress and thereby increasing the risk of POD.

### 3.2 Intraoperative factors

#### 3.2.1 Surgical approach and duration

Surgical approach and operative duration are critical factors significantly influencing the risk of POD (Edwards et al., 2021). Evidence indicates that emergency surgery is a major precipitating factor for POD, with higher incidence rates compared to elective procedures (Liu et al., 2022). Cardiac surgeries involving cardiopulmonary bypass are particularly high-risk, with POD incidence ranging from 37 to 52%, attributable to the combined effects of hemodynamic fluctuations, microemboli formation, and systemic inflammatory responses. Orthopedic major surgeries (~11%) and abdominal procedures (5–51%) pose comparatively lower, yet still notable, risks (Kumar et al., 2017; van Norden et al., 2021). Notably, minimally invasive techniques, such as transcatheter aortic valve implantation (TAVI), substantially reduce surgical trauma; however, POD incidence in elderly patients remains 14.3–18.8%, underscoring advanced age as an independent, non-negligible risk factor (Lee et al., 2023). Patients undergoing major cardiac or large vessel procedures exhibit higher susceptibility to POD than those receiving general surgical operations. Among non-cardiac procedures, thoracic and abdominal surgeries are associated with relatively elevated POD incidence. The high prevalence of POD following major cardiac surgeries is likely linked to systemic inflammatory responses induced by cardiopulmonary bypass (Meco et al., 2023).

#### 3.2.2 Intraoperative blood loss

Intraoperative blood loss has been identified as a critical risk factor for POD. Excessive blood loss or overzealous fluid administration can lead to hemodilution, decreased hemoglobin levels, and reduced hematocrit, impairing the oxygen-carrying capacity of blood and systemic oxygen delivery. Consequently, cerebral oxygen content declines, accompanied by reduced acetylcholine levels in the brain, thereby increasing the risk of POD. Clinical studies have indicated that a hematocrit below 30% may compromise cerebral oxygenation and elevate POD risk. Furthermore, patients experiencing substantial intraoperative blood loss—defined as more than 1,000 mL, postoperative hemoglobin <11 g/dL, or requiring transfusion exceeding 2000 mL—demonstrate a significantly higher incidence of POD, Additionally, a multivariate analysis study on the risk factors for postoperative delirium confirmed that an intraoperative blood loss exceeding 1,000 mL was an independent risk factor for postoperative delirium (Hokuto et al., 2020). Blood transfusions themselves may exacerbate the risk by inducing systemic inflammatory responses, increasing circulating cytokines such as TNF-*α*, IL-6, and IL-10, and generating reactive oxygen species, which collectively promote neuroinflammation and microcirculatory dysfunction (Kunz et al., 2020).

#### 3.2.3 Intraoperative hypoxemia

The intraoperative application of rSO₂ monitoring offers a novel approach to prevent cerebral hypoperfusion. Intraoperative hypoxemia has been strongly associated with the development of POD. Near-infrared spectroscopy (NIRS) studies have demonstrated that prolonged intraoperative rSO₂ reductions—either below 80% of baseline or absolute values <60%—are linearly correlated with increased POD risk. A recent meta-analysis demonstrated that anesthesia management guided by regional rSO₂ monitoring significantly reduced the incidence of POD. This study evaluated POD outcomes across six randomized controlled trials (RCTs) involving 826 patients, with an overall POD incidence of 18% (11.9% in the intervention group vs. 23.8% in the control group). Compared with the control group, the rSO₂ monitoring-guided group had a markedly lower POD incidence (odds ratio [OR] = 0.28; 95% confidence interval [95%CI] = 0.09–0.84; *p* = 0.02; I^2^ = 81%) (Tian et al., 2021).

#### 3.2.4 Intraoperative blood pressure fluctuations

Elderly patients often present with cerebrovascular stiffness and commonly have comorbidities such as hypertension, diabetes, and prior cerebral infarction. Age-related cerebral functional decline and impaired cerebrovascular autoregulation increase susceptibility to hypoperfusion when mean arterial pressure (MAP) falls below the autoregulatory range, thereby elevating the risk of cerebral injury and POD (Lie et al., 2021). In a cohort study enrolling 605 patients, sustained intraoperative hypotension (MAP ≤65 mmHg) for more than 5 min was identified as a factor associated with a significantly elevated risk of postoperative delirium in elderly patients (OR = 3.93; 95%CI = 2.07–7.45; *p* < 0.001) (Duan et al., 2023). When the mean arterial pressure drops below the lower limit of cerebral autoregulation, cerebral hypoperfusion may induce cognitive impairment, as well as deficits in attention and memory—core clinical manifestations of delirium. Conversely, intraoperative hypertension can activate the sympathetic nervous system while suppressing parasympathetic activity, reducing acetylcholine secretion, triggering neuroinflammation, and causing neuronal injury, all of which contribute to POD development. Interestingly, preoperative use of calcium channel blockers has been associated with a higher POD risk compared with renin-angiotensin system inhibitors, yet a lower risk compared with *β*-blockers (Harrison et al., 2020).

#### 3.2.5 Intraoperative hyperventilation

Intraoperative hyperventilation, which lowers end-tidal carbon dioxide (ETCO₂) and arterial CO₂ partial pressure, can induce hypocapnia, leading to cerebral vasoconstriction, reduced cerebral blood flow, and altered cerebral metabolism. These pathophysiological changes have been identified as significant risk factors for POD, A recent cohort study enrolling 71,717 patients reported a dose-dependent association between intraoperative hypocapnia and an elevated risk of postoperative delirium, with hypocapnia identified as a high-risk factor for delirium within 7 days postoperatively (ORadj = 1.77; 95%CI = 1.30–2.41; *p* < 0.001) (Ahrens et al., 2023). Hypocapnia impairs cerebral oxygenation and cerebral perfusion, such that a longer duration and greater severity of intraoperative hypocapnia correlate with a significantly higher risk of subsequent postoperative delirium.

#### 3.2.6 Anesthetic depth

Regarding anesthetic depth, recent evidence suggests that maintaining a bispectral index (BIS) between 45 and 60 may be preferable to deep anesthesia (BIS < 40). While lighter anesthesia (BIS > 60) can reduce drug exposure, it may increase the risk of intraoperative awareness, heightened pain perception, and amplified stress responses to surgical stimuli. Such intraoperative awareness has been associated with impaired cognitive function and negative emotional outcomes (Aceto et al., 2020a,b). Conversely, deep anesthesia may reduce early postoperative pain but can induce burst suppression in the brain, thereby increasing the risk of POD (Evered et al., 2021; Long et al., 2022). Studies have reported that elderly and frail patients are more susceptible to intraoperative burst suppression, and the occurrence of intraoperative burst suppression is associated with an increased risk of postoperative delirium (OR = 4.954; 95%CI = 1.034–23.736; *p* = 0.045). Notably, such patients predominantly present with hyperactive delirium in the postoperative period (Ren et al., 2023).

#### 3.2.7 Temperature management

Intraoperative hypothermia is a common occurrence. Low body temperature can compromise vital signs, impair coagulation, induce arrhythmias, and even precipitate cardiac arrest. Moreover, studies have reported that hypothermia may promote microthrombus formation and induce cerebral vasoconstriction, thereby reducing cerebral blood flow and impairing brain perfusion—factors associated with an increased risk of POD (Ju et al., 2023). Maintaining normothermia is fundamental to physiological homeostasis, and perioperative temperature protection should be a clinical priority.

### 3.3 Postoperative factors

Postoperative prevention of POD is crucial, with pain management being a key component. Pain is a well-established risk factor for POD (Denny and Lindseth, 2020). Numerous studies have reported that higher levels of postoperative pain are associated with increased POD incidence (Mossie et al., 2022). Pain can directly trigger a stress response, leading to elevated serum cortisol levels that inhibit hippocampal function and precipitate delirium. Under stress, increased secretion of adrenaline and noradrenaline elevates cerebral blood flow and oxygen consumption, disrupts neuronal information exchange, and further increases the risk of delirium. Excessive use of opioids for analgesia may also contribute to POD by activating central Toll-like receptor 4 (TLR4) and mediating neuroinflammation (Muscat et al., 2023). High-dose opioids can increase dopaminergic and glutamatergic activity while reducing cholinergic signaling, thereby promoting POD (Liu et al., 2025). Comparative studies indicate that multimodal analgesic strategies—combining regional nerve blocks with nonsteroidal anti-inflammatory drugs (NSAIDs)—can reduce POD incidence compared with opioid-only intravenous regimens (Berger-Estilita and Bilotta, 2025).

Studies have demonstrated an association between opioids and POD. Meperidine, in particular, has been linked to a higher incidence of POD, likely because its metabolites possess anticholinergic properties, which can more readily precipitate delirium. In contrast, the relationship between other opioids such as fentanyl, sufentanil, and hydromorphone and POD remains unclear (Duprey et al., 2021). Some evidence suggests that the route of opioid administration may influence POD risk, with intravenous administration being more likely to induce delirium than oral administration. Sedative agents are also recognized as significant contributors to POD. Propofol and benzodiazepines, including midazolam, have been reported to increase the incidence of postoperative delirium (Tomimaru et al., 2020). Although benzodiazepines are effective for anxiety relief and sedation and can be used in withdrawal-associated delirium, they can paradoxically induce delirium themselves, necessitating careful monitoring during their use (Cui et al., 2020). Notably, patients who discontinue benzodiazepines preoperatively appear to be at higher risk for POD compared with those who continue or have never used these agents (Omichi et al., 2021). Current clinical guidelines do not recommend the use of benzodiazepines for prevention or treatment of POD in elderly or critically ill patients (Söhle and Coburn, 2024).

ICU patients often exhibit disruptions in the sleep–wake cycle, with the majority experiencing sleep disturbances, reduced total sleep time, and alterations in sleep architecture. Recent evidence identifies these disruptions as independent risk factors for POD. Circadian rhythm disturbances can suppress melatonin secretion, impair synaptic plasticity, and directly hinder cognitive recovery (Song et al., 2021). In addition, environmental factors such as lighting and equipment noise, as well as iatrogenic factors including indwelling catheters, physical restraints, and certain medications (notably benzodiazepines and anticholinergic agents), further increase POD risk by restricting patient mobility and inducing direct neurotoxicity. Prolonged ICU stays have been associated with higher POD incidence, and patients requiring intubation are particularly susceptible. Emerging evidence suggests a potential link between melatonin, derived from the tryptophan-serotonin metabolic pathway, and the development of delirium, although the precise mechanism remains unclear (Pandharipande et al., 2010). Sleep fragmentation may also elevate POD risk; patients dependent on monitoring devices or those with obstructive sleep apnea experience frequent sleep interruptions, oxygen desaturations, and arousals, which can alter sleep structure and the threshold for wakefulness, contributing to delirium onset (Dooijeweerd et al., 2022). Furthermore, patients with severe obstructive sleep apnea exhibit intermittent cerebral hypoperfusion and hypoxia during apneic episodes, which activate oxygen-sensing molecular pathways, increase oxidative stress, and induce chronic inflammation, ultimately raising POD risk (Dooijeweerd et al., 2022). Metabolic disturbances in elderly patients, including electrolyte imbalances and malnutrition, can also precipitate delirium. Patients with low body mass index (BMI) are particularly vulnerable to postoperative stress, exhibiting reduced infection defense and an increased likelihood of developing POD.

## 4 Assessment and prediction tools

### 4.1 Clinical assessment scales

Standardized assessment tools form the foundation for identifying and subtyping POD. The Confusion Assessment Method (CAM) is widely used for rapid and straightforward evaluation of delirium by nurses and non-psychiatric clinicians (Yang et al., 2024). For ICU patients, internationally recognized tools such as the CAM-ICU and the Intensive Care Delirium Screening Checklist (ICDSC) are particularly suitable, especially for intubated patients, due to their ease of use (<2 min) and high reliability (Bao et al., 2023). Other commonly employed instruments include 3D-CAM, Nu-DESC, DDS, DOS, NEECHAM, CTD, RASS, and their derivatives (Jensen et al., 2025). Among these, CAM, DDS, and Nu-DESC are characterized by high sensitivity and specificity, ease of administration, and applicability across healthcare personnel. Currently, the DSM-V remains the diagnostic gold standard for POD, though it requires specialized psychiatric evaluation due to its complexity (Xue et al., 2025). Recent improvements in assessment scales have focused on geriatric specialization and symptom quantification. The 4AT scale (rapid assessment test) integrates four domains—alertness, attention, cognitive change, and level of consciousness—and can be administered by non-specialist healthcare staff, making it especially suitable for general ward screening (Tieges et al., 2021). The Memorial Delirium Assessment Scale (MDAS) uses a 0–3 scoring system across 10 symptoms, allowing sensitive detection of subtle changes and is suitable for efficacy evaluation and long-term follow-up. MDAS and DRS-R-98 are applicable to all patients, while CAM-S and 3D-CAM-S are tailored for non-ICU populations; all of these scales can assess POD severity. Postoperative delirium shares certain behavioral features with postoperative agitation. With advances in research, more refined methods for differentiating the two have been developed. The European Society of Anaesthesiology strongly recommends evaluating delirium starting from the anesthetic emergence phase and has introduced the concept of emergence delirium (ED), defined as POD occurring in the operating room or post-anesthesia care unit (PACU) immediately after anesthesia (Zhang Y. et al., 2020). Timely assessment is typically conducted using instruments such as the Nursing Delirium Screening Scale (Nu-DESC), ICDSC, and CAM-ICU.

### 4.2 Biomarker-based predictive models

Incorporating biomarkers into predictive models has significantly enhanced the accuracy and timeliness of POD risk assessment, facilitating the prediction of onset, evaluation of severity, and understanding of prognosis, while also providing insights into underlying mechanisms (Wan et al., 2025). Currently, biomarkers associated with POD include inflammatory mediators, cholinergic markers, genetic loci, neurotransmitters, and indicators of brain injury. Inflammatory cytokines such as IL-6, IL-1*β*, IL-8, IL-10, TNF-α, CRP, neutrophil gelatinase-associated lipocalin (NGAL), and STREM2 have been shown to correlate with POD incidence (Khan et al., 2022; Imai et al., 2023; Ruhnau et al., 2023). Combined detection of inflammatory markers (e.g., IL-6 + CRP), neurotrophic factors (BDNF), and brain injury markers (S100β) can identify high-risk states 24–48 h before the onset of clinical symptoms (Ruhnau et al., 2023; Ruhnau et al., 2025). Several studies have identified S100*β* as an independent biomarker for POD (Lai et al., 2023). S100*β*, predominantly secreted by astrocytes, is a calcium-binding protein that increases blood–brain barrier permeability, and its expression is elevated in POD patients (Zhang X. et al., 2020). Alterations in the ratio of β-amyloid to Tau proteins are also associated with POD onset (Subramaniyan and Terrando, 2019). Tau, a neurodegenerative protein linked to dementia, stroke, and traumatic brain injury, has been found at higher concentrations in delirium patients, correlating with the severity of POD (Ballweg et al., 2021). Phosphorylated Tau (pTau) represents a novel biomarker for POD; in aged delirium mouse models, elevated levels of Tau-PT217 and Tau-PT181 have been detected, providing new avenues for POD research (Liang et al., 2023). CRP is another biomarker predictive of POD risk; patients with higher CRP levels have a 1.5-fold increased risk of developing POD compared with those with lower levels (Vasunilashorn et al., 2015). Serum neurofilament light chain (NfL) serves as a marker of axonal injury, which may disrupt neural network connectivity and contribute to POD pathogenesis (Fong et al., 2020). Perioperative probiotic interventions have been shown to reduce IL-1β and IL-6 levels while increasing BDNF, significantly improving cognitive outcomes, and highlighting a biomarker-guided intervention strategy (Lee et al., 2021). Additionally, TAR DNA-binding protein 43 (TDP-43), a pathological marker in frontotemporal dementia, and mitochondrial YARS2, which is elevated in aging and cognitive dysfunction, have been implicated in neurodegenerative processes (Lye and Chen, 2022; Jhanji et al., 2022). Collectively, emerging evidence suggests that a diverse array of biomarkers may play a critical role in predicting POD and elucidating its underlying mechanisms in future research.

### 4.3 Electronic early warning systems and artificial intelligence

Traditional early warning scores (EWS) have shown limited predictive performance in elderly patients. To address these limitations, continuous wireless monitoring technologies and AI-based early warning systems have emerged as research priorities. By continuously collecting multidimensional physiological data—such as heart rate variability, body movements, and respiratory patterns—and analyzing pattern changes with deep learning algorithms, these systems can detect subclinical alterations in advance. AI-driven predictive models demonstrate significantly improved sensitivity in identifying high-risk patients, markedly outperforming conventional approaches.

### 4.4 Electroencephalography and neuromuscular monitoring

Electroencephalography (EEG), by recording the electrical activity of cortical neurons, is highly sensitive to cerebral ischemia and hypoxia and can detect early changes in brain function. Preoperative reductions in EEG β- and *γ*-band power have been identified as biomarkers indicative of increased POD risk (Schüßler et al., 2023), while intraoperative decreases in *α*- and β-band power serve as predictive EEG markers for POD (Acker et al., 2024; Ostertag et al., 2024). Currently, processed EEG metrics, such as the BIS, are widely employed to monitor anesthetic depth, and preventing intraoperative burst suppression has been shown to reduce POD incidence. Studies have also reported a dose-dependent relationship between intraoperative administration of neuromuscular blocking agents and POD risk. Utilizing neuromuscular monitoring allows for minimizing the dosage of muscle relaxants while ensuring adequate surgical relaxation, thereby contributing to the prevention of POD (Ahrens et al., 2025).

## 5 Comprehensive intervention strategies

POD in elderly patients can significantly impair quality of life, prolong hospital stays, and increase healthcare costs. Moreover, elderly patients who develop POD face a higher risk of mortality, underscoring the critical importance of prevention. Substantial evidence indicates that approximately 30–40% of POD cases are potentially preventable (Fenta et al., 2025). Implementing targeted preventive strategies based on identified risk factors can effectively reduce the incidence and severity of POD in older adults (Baek et al., 2020).

### 5.1 Preoperative optimization strategies

Comprehensive Geriatric Assessment (CGA) is a multidisciplinary, systematic approach designed to evaluate the overall status of elderly patients and address their usual and complex needs, serving as a critical tool for preoperative optimization. CGA encompasses multidimensional assessments of nutritional status, cognitive function, medication management, and social support systems, which form the basis for developing individualized intervention plans (Zietlow et al., 2022). Evidence suggests that CGA-based perioperative care can significantly reduce the risk of postoperative delirium, lowering the absolute incidence to 8.28% (95% CI = 3.9–12.6) (Saripella et al., 2021). Emerging strategies such as probiotic supplementation have shown promise in POD prevention. The proposed mechanisms include suppression of proinflammatory cytokines IL-1β and IL-6, enhancement of BDNF levels, and modulation of gut retinoid metabolic pathways, collectively contributing to improved neurocognitive resilience.

### 5.2 Intraoperative protective measures

The rSO₂ monitoring represents a fundamental strategy for intraoperative brain protection. Maintaining rSO₂ above 90% of baseline is recommended and can be achieved by optimizing hemoglobin levels (>9 g/dL), controlling PaCO₂ within the upper normal range (40–45 mmHg), and judicious use of vasoactive agents. Intraoperative anesthesia depth monitoring can further guide anesthetic management. Maintaining BIS values within 45–60 helps avoid excessively deep anesthesia (BIS <40) and prevents intraoperative EEG burst suppression, thereby reducing the risk of postoperative delirium. Evidence indicates that BIS-guided anesthesia management effectively decreases the incidence of POD within the first 7 days following non-cardiac surgery.

Intraoperatively, hypotension and marked blood pressure fluctuations should be avoided. Goal-directed fluid therapy combined with norepinephrine can be employed for intraoperative circulatory management (Yoshikawa et al., 2024). The choice of intravenous fluids is also relevant; studies indicate that colloid solutions are more likely than crystalloids to increase the risk of POD, with hydroxyethyl starch (HES) posing a higher risk than human albumin. This difference may relate to the anti-inflammatory properties of albumin, which can reduce neuroinflammation, whereas HES may contribute to subclinical cerebral edema. Additionally, persistent intraoperative low end-tidal CO₂ (PetCO₂) and the severity of its reduction have been associated with POD severity, likely due to decreased cerebral blood flow. Employing lung-protective ventilation strategies—including low tidal volumes, low levels of positive end-expiratory pressure, appropriate respiratory rates, and alveolar recruitment—can increase arterial CO₂, reduce blood pH, enhance cerebral oxygen delivery, mitigate inflammatory responses, and ultimately decrease the incidence of postoperative delirium.

The choice of anesthetic technique requires careful consideration of benefits and risks. Regional anesthesia has demonstrated advantages in hip and knee arthroplasty; compared with general anesthesia, patients receiving regional anesthesia exhibit higher postoperative MMSE scores within 7 days and significantly lower stress indicators such as cortisol and blood glucose. Perioperative low-dose intravenous corticosteroids may help reduce POD incidence, potentially by suppressing trauma-induced inflammatory responses (An et al., 2023). Preoperative administration of dexamethasone can modulate the hypothalamic–pituitary–adrenal axis via negative feedback, reducing stress-induced cortisol secretion, improving sleep quality, preventing nausea and vomiting, mitigating allergic reactions, and decreasing recruitment of astrocytes and microglia while attenuating inflammatory cytokine expression, thereby exerting anti-inflammatory effects that may lower POD risk (Peles et al., 2023). Regarding anesthetic maintenance, sevoflurane use has been associated with a lower risk of POD compared with isoflurane, desflurane, and propofol (Taschner et al., 2024). Large-scale studies also indicate that propofol anesthesia results in lower POD incidence within 5 days postoperatively than sevoflurane anesthesia (Cao et al., 2023). Recently, esketamine has shown potential neuroprotective effects through competitive NMDA receptor antagonism, AMPA receptor activation, and upregulation of BDNF, while improving cognitive function, mitigating stress responses, reducing inflammation, and demonstrating high clearance with few side effects, suggesting promise as a POD therapeutic agent (Wei et al., 2020; Araújo-de-Freitas et al., 2021). In elderly patients undergoing hip fracture surgery, continuous intraoperative intravenous lidocaine infusion has been shown to reduce POD incidence and lower the occurrence of intraoperative hypertension and tachycardia (Li et al., 2023). However, recent large-scale studies suggest that the independent effect of anesthesia type on POD may be less significant than the overall quality of perioperative management. Most antipsychotics and sedatives carry a risk of inducing POD and should be used cautiously (Banning et al., 2021). Additionally, intraoperative administration of tropisetron may reduce the incidence of POD in non-cardiac surgery patients (Sun et al., 2020).

Strict infection prevention and control should be implemented, and homeostasis should be maintained in elderly patients by ensuring proper fluid-electrolyte balance, acid–base equilibrium, and overall internal environment stability. Unnecessary invasive devices, such as nasogastric or urinary catheters, should be minimized. Intraoperative vital signs must be closely monitored, including oxygen saturation, to prevent hypoxemia and cerebral ischemia or hypoxia. Temperature management is also crucial, as hypothermia can lead to adverse effects and increase the risk of postoperative delirium.

### 5.3 Postoperative multimodal interventions

Early postoperative interventions for elderly patients include pain management, spontaneous breathing trials, careful sedative selection, delirium screening, early mobilization, and family involvement. POD interventions can be broadly classified into non-pharmacological and pharmacological approaches (Liu et al., 2022), with current guidelines primarily recommending non-pharmacological strategies. Evidence suggests that cognitive training, family presence, familiar environmental cues, cognitive-behavioral support, music therapy, and massage can effectively improve POD clinical symptoms, reduce delirium severity, and shorten its duration (Burton et al., 2021; Hosie et al., 2021).

In elderly patients, preoperative avoidance of diazepam and barbiturates is recommended, and caution should be exercised with intraoperative use of benzodiazepines and anticholinergic drugs such as midazolam and pentazocine, as these medications may increase the risk of POD (Yang et al., 2023). However, studies have reported that remimazolam is not associated with POD within the first five postoperative days, likely due to its ultra-short-acting benzodiazepine properties; it is rapidly hydrolyzed by hepatic carboxylesterases, exhibiting high clearance, small volume of distribution, and a shorter half-life than midazolam (Aoki et al., 2023). For patients who develop POD, low-dose dexmedetomidine can effectively and rapidly alleviate symptoms, and evidence suggests it may also have prophylactic effects. Dexmedetomidine is a highly selective α2-adrenergic receptor agonist that provides sedation, analgesia, and anxiolysis while suppressing sympathetic overactivity (van Norden et al., 2021). It activates the vagus nerve, reduces blood pressure and heart rate, decreases myocardial oxygen consumption, and promotes near-physiological sleep cycles. Additionally, dexmedetomidine mitigates neuroinflammation, activates anti-apoptotic pathways, and stabilizes central hemodynamics, thereby exerting neuroprotective effects (Berger-Estilita and Bilotta, 2025). Prophylactic administration of low-dose dexmedetomidine has been shown to significantly reduce the incidence of POD within 7 days postoperatively in non-cardiac surgical patients aged ≥65 years (Momeni et al., 2021). Haloperidol is also effective for rapid treatment of POD. As a butyrophenone antipsychotic, it blocks dopamine D2 receptors, maintains acetylcholine-dopamine balance in the central nervous system, attenuates inflammatory injury to hippocampal neurons, reduces neurotransmission disturbances, and protects cognitive function (Castro et al., 2020). Haloperidol exerts anxiolytic, antipsychotic, and anti-agitation effects (Siegel et al., 2023), although its extrapyramidal and anticholinergic side effects warrant careful consideration (Simon et al., 2025). Elderly patients with both dementia and POD exhibit worse outcomes than those with dementia alone, including faster cognitive decline, prolonged hospitalization, and increased mortality. Perioperative melatonin administration can improve circadian rhythm and reduce the incidence of POD in elderly patients. Administration of a higher dose of melatonin (5 mg) within five elimination half-lives before surgery has been suggested to be beneficial for POD prevention (Han et al., 2020). Postoperative multimodal analgesia is recommended. Studies indicate that the NSAID parecoxib sodium effectively inhibits cyclooxygenase-2 activity, significantly alleviating postoperative pain (Daojun et al., 2025). It also reduces the risk of POD by suppressing inflammation and oxidative stress (Cozowicz et al., 2021). Moreover, parecoxib exerts anti-inflammatory effects within the central nervous system, decreases reactive oxygen species and prostaglandins, mitigates stress responses, and improves local cerebral microcirculation, thereby lowering the risk of delirium.

**Figure 2:**
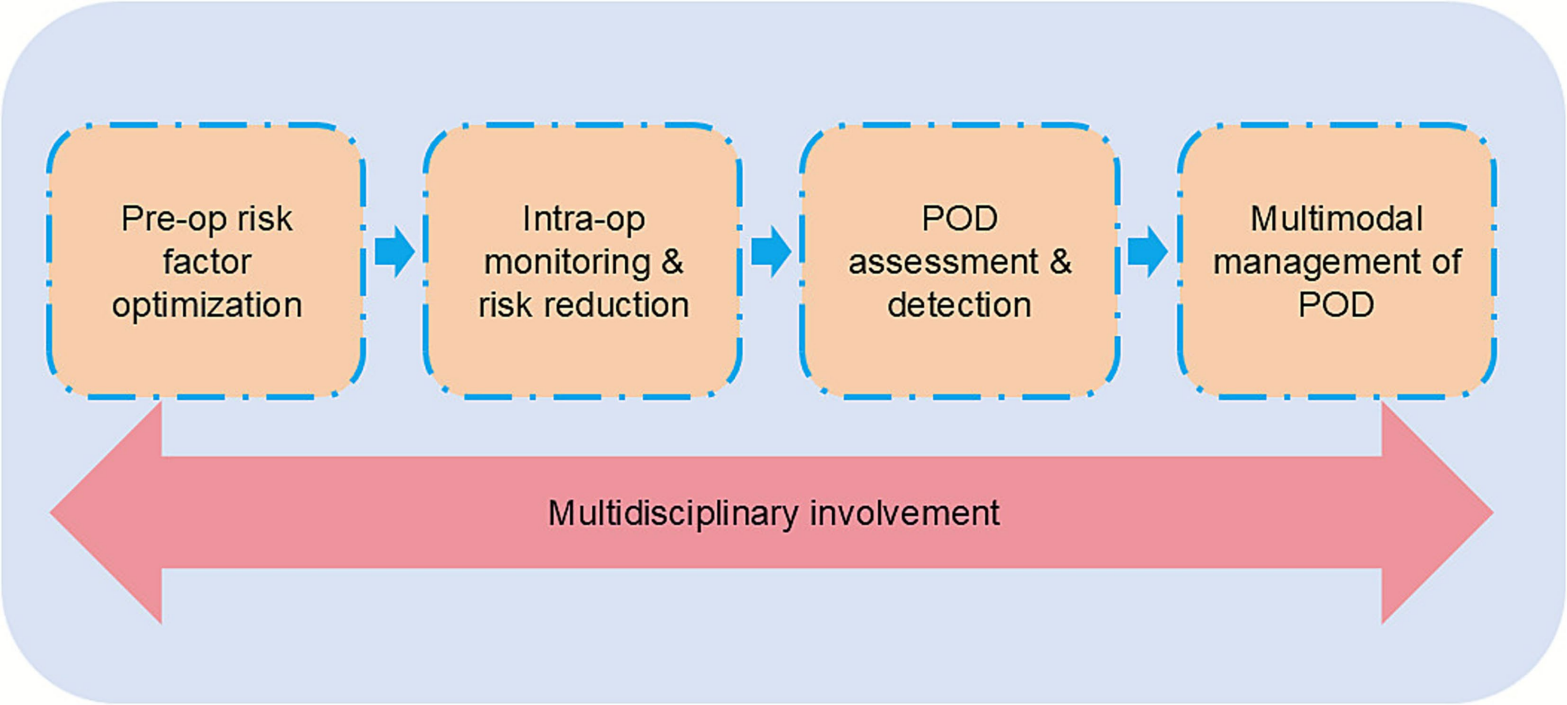
Multidisciplinary perioperative management framework for POD. The schematic illustrates a comprehensive approach to POD prevention and management, encompassing four sequential stages: (1) Preoperative risk factor optimization, (2) intraoperative monitoring and risk reduction, (3) POD assessment and detection, and (4) multimodal management of POD. Continuous multidisciplinary involvement, represented by the large arrow, underlies all stages, emphasizing coordinated care across healthcare professionals to minimize the incidence and severity of POD.

Pain not only activates glial cells but also elevates endogenous inflammatory mediators, leading to neuronal dysfunction and contributing to the development of POD. Effective pain management can therefore reduce the risk of POD. Intraoperative nerve blocks, either alone or combined with general anesthesia, can significantly decrease perioperative opioid consumption, provide adequate postoperative analgesia, alleviate pain, and facilitate early mobilization, thereby promoting recovery and reducing the incidence of POD (see Figure 2).

## 6 Summary and outlook

With the advent of an aging society, the health challenges faced by older adults are increasing, and a growing number of elderly patients are undergoing surgical procedures. The high incidence of POD in this population has drawn considerable attention. POD is a common multifactorial syndrome in older surgical patients and is closely associated with adverse outcomes. Research has shifted from identifying individual risk factors to exploring multi-system interactions, and from universal interventions to precision prevention strategies. Current evidence highlights neuroinflammation, neurotransmitter imbalance, and disruption of neural networks as central pathological mechanisms, while perioperative frailty, imbalances in cerebral oxygen supply and demand, and disturbances in the sleep–wake cycle are key risk factors. Multidimensional assessment tools combined with staged intervention strategies provide practical approaches to address these challenges.

The gut–brain axis may represent a critical pathway underlying the development of POD in elderly patients. Elucidating how microbial metabolites regulate blood–brain barrier permeability and microglial polarization can enhance our understanding of the gut–brain axis in POD pathophysiology. Additionally, integrating multi-omic biomarkers into machine learning-based dynamic risk prediction models may more accurately identify high-risk populations, providing targeted guidance for prevention and intervention. POD arises from the interplay of multiple factors; for high-risk patients, preoperative interventions—such as correction of frailty, nutritional optimization, vitamin D supplementation, probiotics, and melatonin administration—combined with rigorous intraoperative monitoring of vital signs, cerebral oxygenation, and anesthetic depth, followed by postoperative multimodal analgesia and enhanced non-pharmacological strategies including family engagement, can significantly reduce the incidence of POD in elderly surgical patients.

Achieving these goals requires deep interdisciplinary collaboration among perioperative medicine, geriatric medicine, and neuroscience. Preventing POD in elderly patients is of critical importance, as no definitive or highly effective treatment currently exists. Therefore, mitigating risk factors remains the most effective strategy to control POD. Only through cross-disciplinary cooperation and evidence-based clinical practice can we advance the understanding of POD, reduce its incidence, enhance perioperative patient safety, and ultimately improve the quality of life of elderly surgical patients, addressing the challenges posed by an aging surgical population.
